# Supplementary material for: A Facile Graphene Conductive Polymer Paper Based Biosensor for Dopamine, TNF-α, and IL-6 Detection
Source: Sensors (Basel). 2023 Sep 27;23(19):8115. doi: 10.3390/s23198115 (PMC10575219; doi:10.3390/s23198115)
Supplement: Supplementary file 1 [file sensors-23-08115-s001.zip › sensors-2598240-supplementary.pdf]

sensors

Supplementary

Article

A Facile Graphene Conductive Polymer Paper Based Biosensor for Dopamine, TNF-α, and IL-6 Detection

Md Ashiqur Rahman [1], Ramendra Kishor Pal [2], Nazmul Islam [3], Robert Freeman [4], Francois Berthiaume [5], Aaron Mazzeo [6,*] and Ali Ashraf [4,*]

[1] Department of Mechanical Engineering, Purdue University, West Lafayette, IN 47906, USA; rahma144@purdue.edu
[2] Hyderabad Campus, Birla Institute of Technology and Science Pilani, Hyderabad 500078, Telangana, India; ramendra.pal@hyderabad.bits-pilani.ac.in
[3] Department of Electrical and Computer Engineering, University of Texas Rio Grande Valley, Edinburg, TX 78539, USA; nazmul.islam@utrgv.edu
[4] Department of Mechanical Engineering, University of Texas Rio Grande Valley, Edinburg, TX 78539, USA; robert.freeman@utrgv.edu
[5] Department of Biomedical Engineering, Rutgers University, Piscataway, NJ 08854, USA; fberthia@soe.rutgers.edu
[6] Department of Mechanical & Aerospace Engineering, Rutgers University, Piscataway, NJ 08854, USA
* Correspondence: aaron.mazzeo@rutgers.edu (A.M.); ali.ashraf@utrgv.edu (A.A.)

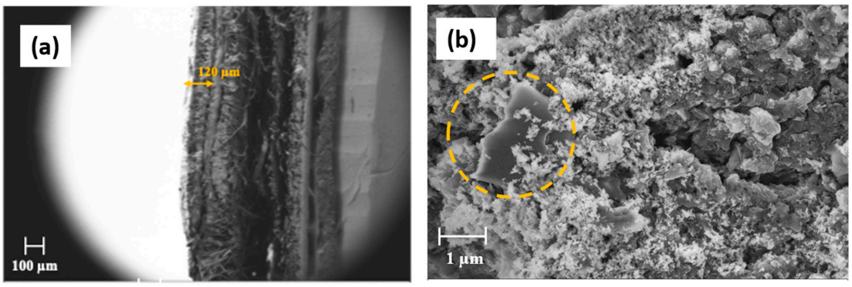

Figure S1. Morphological characterizations at lower scale. Scanning Electron Microscopy (SEM) characterization of G-PEDOT:PSS ink at a lower magnification of showing (a) the coating thickness of 120 µm, (b) graphene attached to the conductive polymer.

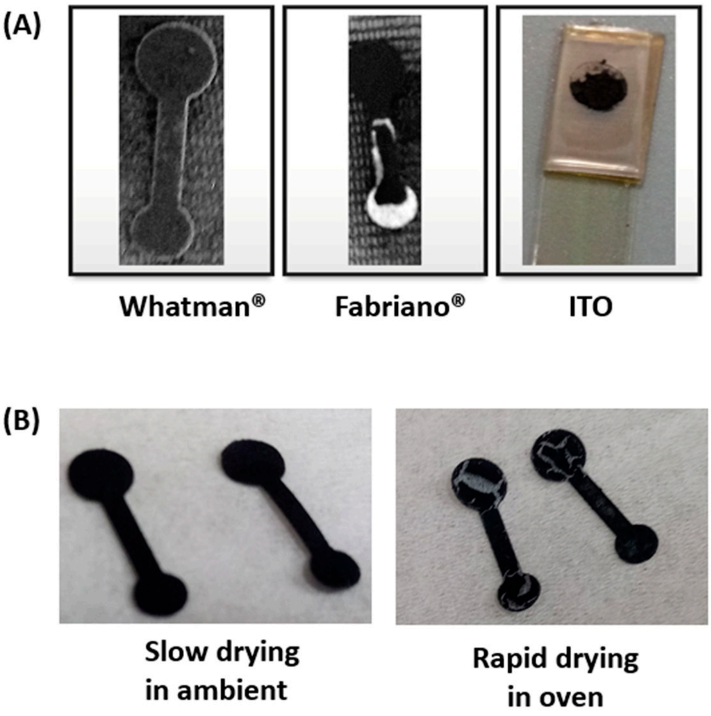

Figure S2. Effect of substrates and drying temperature on film stability. (a) Film stability of GR-CP conductive film on different substrates. The film is stable on Whatman filter paper but delaminates on hydrophobic Fabriano paper and hard ITO substrate. (b) Effect of drying temperature on film stability. Film delaminates if dried at high temperature (~70 C).

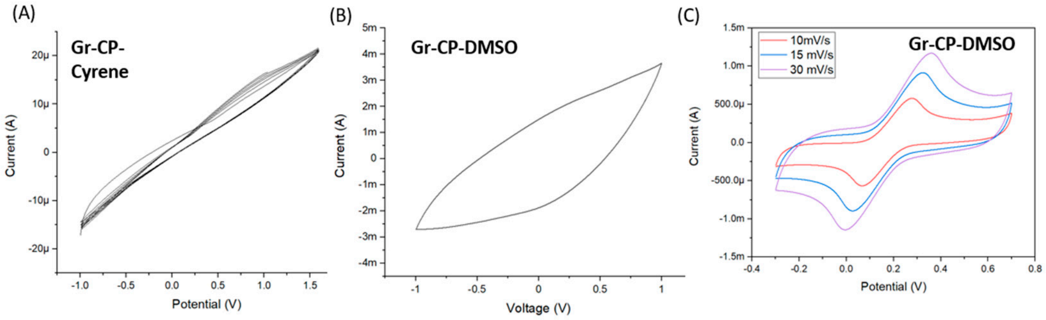

Figure S3. Conductivity test for Cyrene and DMSO solvent. Cyclic voltammetry (CV) of GR-CP ink prepared with (A) Cyrene and (B,C) DMSO. Ink with DMSO show superior conductivity compared to ink with Cyrene. (C) shows CV with different scan rates in 10 mM Ferri-Ferro solution.

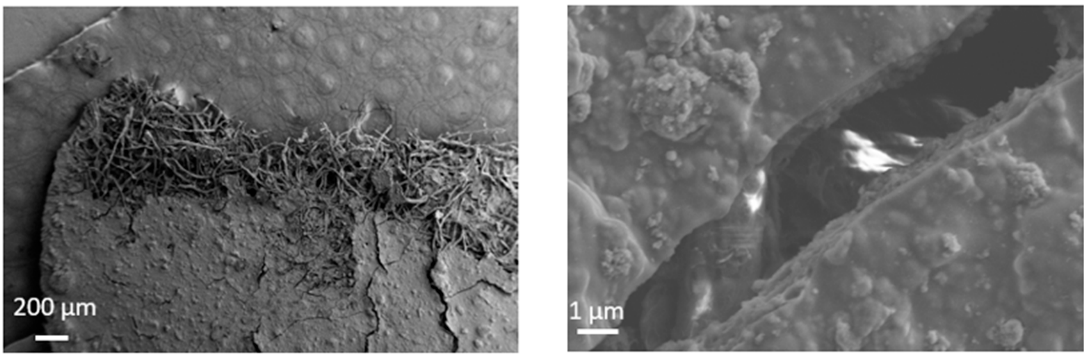

Figure S4. Morphology of thick ink coating. SEM images of sensor prepared with thick ink coating (includes double the amount of PEDOT-PSS as compared to original ink). After drying, forms a thick layer or crust, preventing access to the micro-nanoporous structure underneath except at cracks.

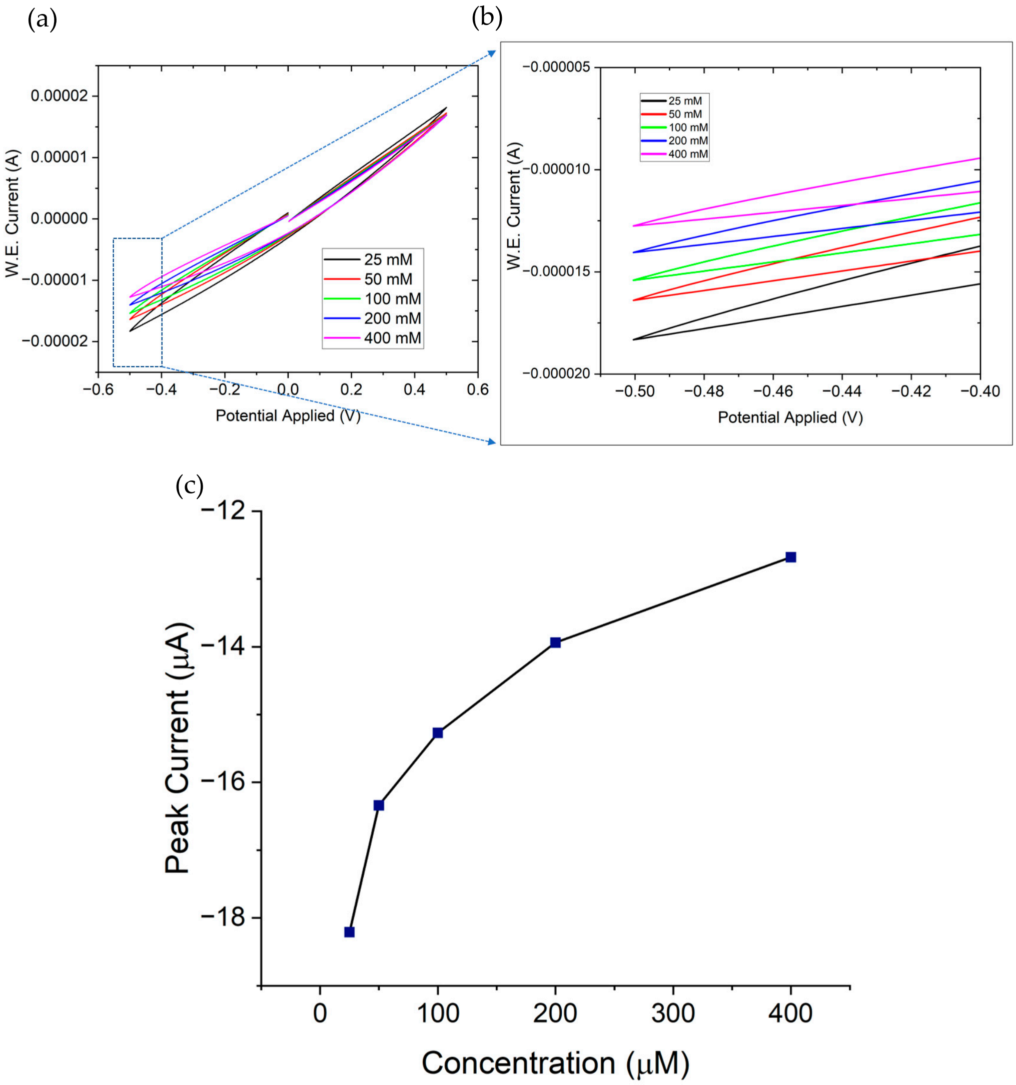

Figure S5. Cyclic Voltammetry performance on single-layer paper-based biosensor. (a) Cyclic Voltammetry (CV) performance for single layer coated paper-based electrodes during dopamine

detection, (b) enlarged view of Figure S5 (a), (c) Peak current-concentration plot of CV performed on paper-based biosensor.

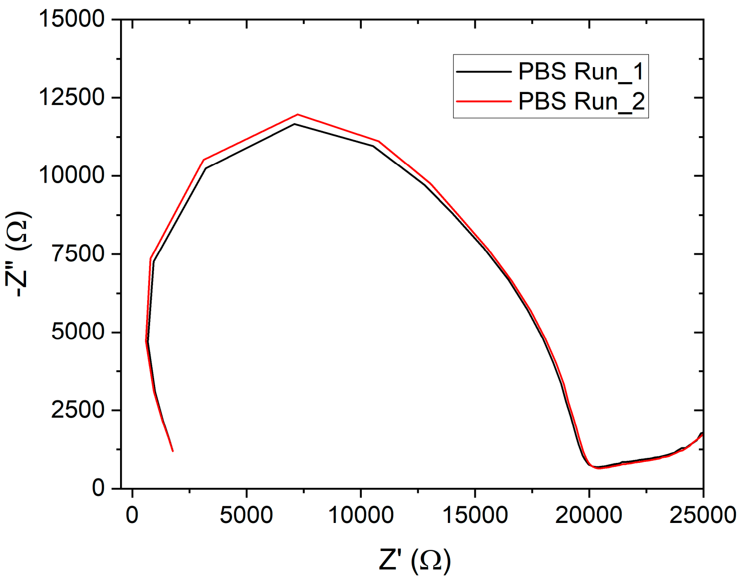

Figure S6. Control run for dopamine detection. Control run test for dopamine detection in paper-based electrodes (10 μL of Fe^{2+}, 10 μL of Fe^{3+}, 2 μL PBS). Charge transfer resistance was similar each time.

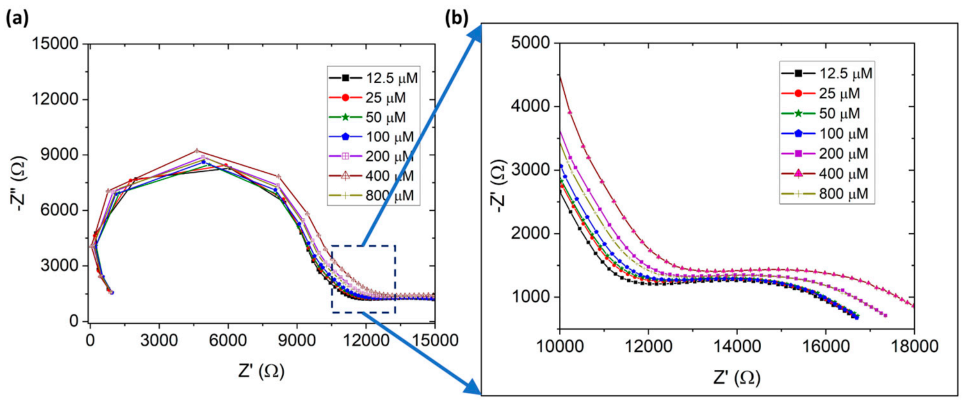

Figure S7: Effect of concentration higher than the saturation. Nyquist plot for dopamine detection from 12.5 μM to 800 μM. The charge transfer resistance increased from 12.5 μM to 400 μM, however, the charge transfer resistance decreased for 800 μM.

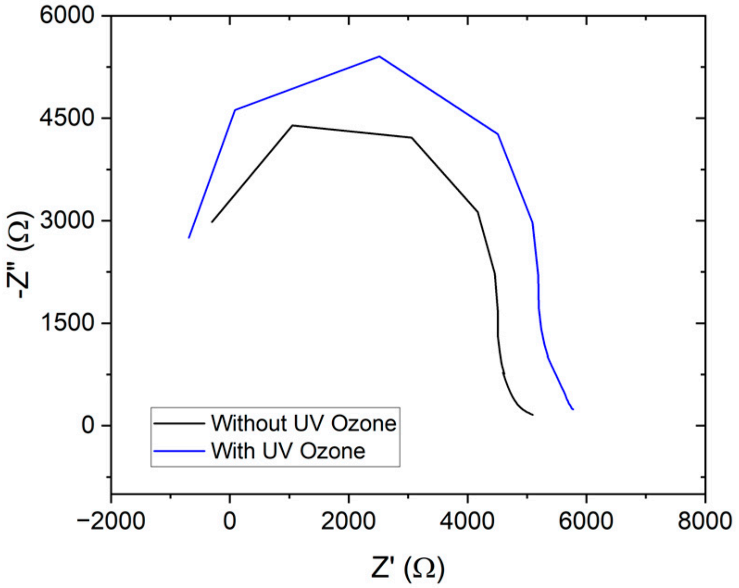

Figure S8: Effect of UV ozonation on paper-based biosensor. Control run test to examine the effect of UV ozonation. The test was done by using 10 mL of Fe^{2+}, 10 mL of Fe^{3+}, and 2 mL of PBS solution before and after UV ozonation. The plot shifted due to surface medication caused by UV ozone (change in resistance).

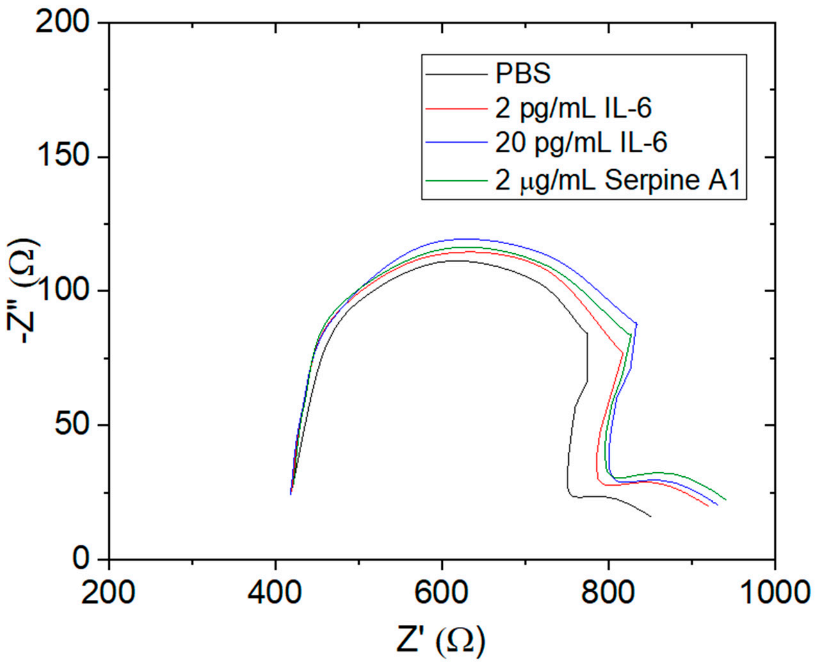

Figure S9: Additional selective detection test. Additional selective detection of IL-6 with Serpin A1 antigen. The charge transfer resistance did not change significantly after the addition of Serpin A1(semicircular portion was added for visual reference).
